# DNA methylation and genetic degeneration of the Y chromosome in the dioecious plant *Silene latifolia*

**DOI:** 10.1186/s12864-018-4936-y

**Published:** 2018-07-16

**Authors:** José Luis Rodríguez Lorenzo, Roman Hobza, Boris Vyskot

**Affiliations:** Plant Developmental Genetics, Institute of Biophysics v.v.i, Academy of Sciences of the Czech Republic, Královopolská 135, 612 65 Brno, Czech Republic

**Keywords:** Epigenetics, Sex chromosomes, *Silene latifolia*, Y degeneration, Sex-linked genes, DNA methylation, Sodium bisulfite, Immunoprecipitation

## Abstract

**Background:**

*S. latifolia* is a model organism for the study of sex chromosome evolution in plants. Its sex chromosomes include large regions in which recombination became gradually suppressed. The regions tend to expand over time resulting in the formation of evolutionary strata. Non-recombination and later accumulation of repetitive sequences is a putative cause of the size increase in the Y chromosome. Gene decay and accumulation of repetitive DNA are identified as key evolutionary events. Transposons in the X and Y chromosomes are distributed differently and there is a regulation of transposon insertion by DNA methylation of the target sequences, this points to an important role of DNA methylation during sex chromosome evolution in *Silene latifolia*. The aim of this study was to elucidate whether the reduced expression of the Y allele in *S. latifolia* is caused by genetic degeneration or if the cause is methylation triggered by transposons and repetitive sequences.

**Results:**

Gene expression analysis in *S. latifolia* males has shown expression bias in both X and Y alleles. To determine whether these differences are caused by genetic degeneration or methylation spread by transposons and repetitive sequences, we selected several sex-linked genes with varying degrees of degeneration and from different evolutionary strata. Immunoprecipitation of methylated DNA (MeDIP) from promoter, exon and intron regions was used and validated through bisulfite sequencing. We found DNA methylation in males, and only in the promoter of genes of stratum I (older). The Y alleles in genes of stratum I were methylation enriched compared to X alleles. There was also abundant and high percentage methylation in the CHH context in most sequences, indicating de novo methylation through the RdDM pathway.

**Conclusions:**

We speculate that TE accumulation and not gene decay is the cause of DNA methylation in the *S. latifolia* Y sex chromosome with influence on the process of heterochromatinization.

**Electronic supplementary material:**

The online version of this article (10.1186/s12864-018-4936-y) contains supplementary material, which is available to authorized users.

## Background

**Fig. 1:**
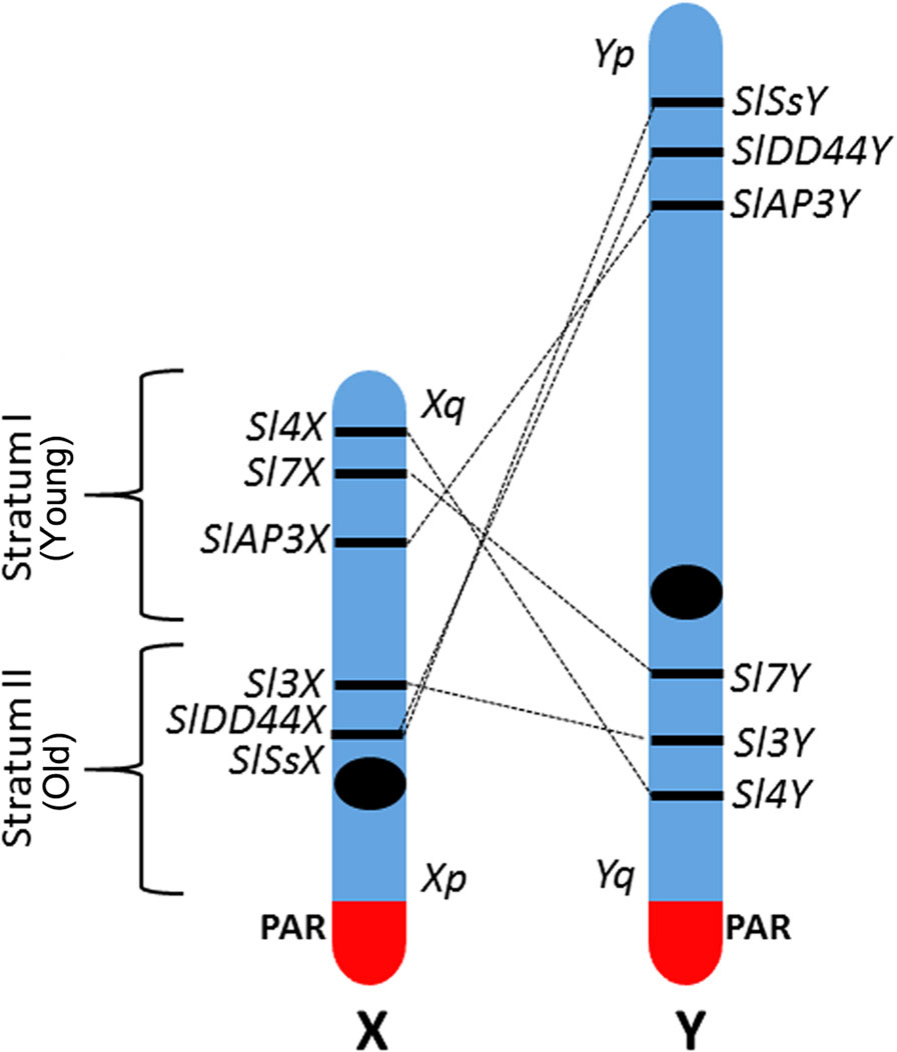
Schematic map (not in scale) of the six X and Y linked alleles under analysis in this study according to Hobza, Kazama and Zluvova [2, 5, 6]. The different gene rearrangement between both chromosomes was caused by regions that underwent chromosomal inversions. Strata distribution in the X chromosome according to Papadopulos [26]

While sex chromosomes in mammals are ancient, those in dioecious plants are evolutionarily young. The plant Y chromosome, which is largely non-recombining, represents a unique part of the genome. In contrast to the small mammalian Y chromosome, heteromorphic Y chromosomes in angiosperms are often the largest chromosomes in the male genome, e.g., in *Silene latifolia* and *Cannabis sativa* [1]. *S. latifolia*, a dioecious plant of the Caryophyllaceae family with sex chromosomes, originated around 10 million years ago. It is a model organism for studying sex chromosome evolution in plants. A distinguishing feature shared by independently evolved sex chromosomes is the presence of a suppressed recombination region on a sex-specific chromosome in the heterogametic sex. The X chromosome in *S. latifolia* is submetacentric and two arms, p and q, can be identified. On the other hand, the Y chromosome is metacentric and it is about 1.4 times larger than the X chromosome. The pseudoautosomal region (PAR) is located in the short subtelomeric region of the p arm on the X chromosome and the q arm of the Y chromosome. The non-recombining region includes at least two sex-determining loci; a gynoecium suppression function (GSF) and a stamen promoting function (SPF) [2, 3]. It has been suggested that *S. latifolia* Y chromosome gradually stopped recombining [4]. Sex chromosome pairs include regions in which recombination became suppressed at different times. These non-recombining regions tend to expand over evolutionary time resulting in the of evolutionary strata formation. The *S. latifolia* X Y sex chromosome pair has at least two strata, with the more recent stratum adjacent to the recombining pseudo-autosomal region (PAR) [3]. According to Kazama and Zluvova [5, 6], there is a large pericentric inversion between X and Y chromosomes. By comparing the Y-linked genes analysed in the presented study with the corresponding X-linked copies (Fig. 1), gene arrangement differences are evident. Cessation of recombination between large parts of the sex chromosomes is the most probable cause of increase in the size of the Y chromosome. Two important processes are involved in non-recombining regions of a genome. The first is accumulation of various types of repetitive DNA sequences such as transposable elements and tandem repeats. The other is gene degeneration [7]. The accumulation of many types of DNA sequence repeats (satellites, microsatellites, plastid sequences, transposons) may play an indirect (epigenetic) role in Y-chromosome decay. Transposons are differently distributed in the X and Y chromosomes of *S. latifolia* [8]. Kubat [9] showed that the Ogre retrotransposon family evolved before the appearance of sex chromosomes but was mobilised after formation of the Y chromosome. These authors suggest that transposons could play a role in sex chromosome evolution in *S. latifolia* through epigenetic silencing mechanisms. Transposon insertion is frequently regulated by the genome itself (e.g. using RNAi machinery), resulting in DNA methylation of target sequences. Methylation spreads to regions surrounding transposons with local effect on the expression of linked genes [10, 11].

In animals, DNA methylation plays an important part in the early stages of sex chromosome evolution and, degeneration of the Y chromosome is linked to epigenetic regulation [12]. Waddinton [13] defined epigenetics as “the branch of biology that studies the causal interactions between genes and their products which bring the phenotype into being”, and can be understood as a system for selectively regulating genome information through activating or inactivating gene expression. DNA methylation can be divided into three types according to the sequence context of the cytosines, namely CG, CHG, and CHH (H = A, C, or T). CG and CHG contexts are symmetrical unlike CHH context which is asymmetrical. The symmetrical nature of CpG context is critical to the maintenance mechanism which is performed by conserved Dnmt1 DNA methyltransferases. The mechanisms of methylation in the mCHG and mCHH contexts are unique to plants [14]. The Methylation levels in each context differ between plant species, with mCG varying ~ 3×, mCHG ~ 9×, and mCHH ~ 16× [15]. The effect of methylation on gene expression is highly dependent upon the type of methylation as well as the pattern of that methylation within or outside the gene [16]. In plants, de novo methylation takes place in a CHH context and is established by transcription of the two RNA polymerases: *POLYMERASE IV* (*Pol IV*) and *Pol V*. The *Pol IV* and *Pol V* transcripts serve as a template for the RNA-dependent RNA polymerase, generating double-stranded RNA which is digested by *DICER-LIKE 3* activity into 24-nt sRNAs. The 24-nt sRNAs, signal *DRM2* to initiate CHH methylation including an Argonaute complex. This mechanism has been described as RNA-directed DNA methylation (RdDM) [17]. The Y chromosome evolution in *Carica papaya* is linked to DNA methylation and heterochromatinisation [18]. This is the mechanism proposed to induce dioecy and the evolution of sex chromosomes, with suppression of meiotic recombination [19]. In *S. latifolia*, DNA methylation is also proposed as the mechanism underlying female sex suppression in the Y chromosome of male individuals [20].

This study is based on the general hypothesis that there is an epigenetic influence on sex determination and evolution of plant sex chromosomes. It is known that most of the alleles in the Y chromosome with existing X linked counterparts characterized in *S. latifolia* so far, have a partial Y expression insufficiency. The lack of recombination in the sex specific region leads to an accumulation of deleterious mutations and degeneration. The reduced effective population size of the Y chromosome is one of the main forces leading to degeneration [7]. Not only gene degradation, but DNA rearrangements and insertions of repetitive sequences and transposons can also affect the transcription of genes in the Y chromosome [2, 21, 22]. The aim of this study was to elucidate whether the reduced expression of the Y alleles is caused by genetic degeneration, or if the cause is methylation spread by transposons and repetitive sequences.

## Results

### Expression results

Expression of the genes *SlAP3/SvAP3; SlDD44/SvDD44; Sl3/Sv3; Sl4/Sv4; Sl7/Sv7; SlSs/SvSs* was measured in tissues at different developmental stages: leaves in vegetative growth, leaves during the flowering stage and flower buds. The flower buds were selected from stage 7 to 10 according to Farbos [23]. These genes belong to stratum I (older) or stratum II (younger) of the X chromosome from the evolutionary point of view (Fig. 1). Full expression results can be found in Additional file 1.

**Fig. 2:**
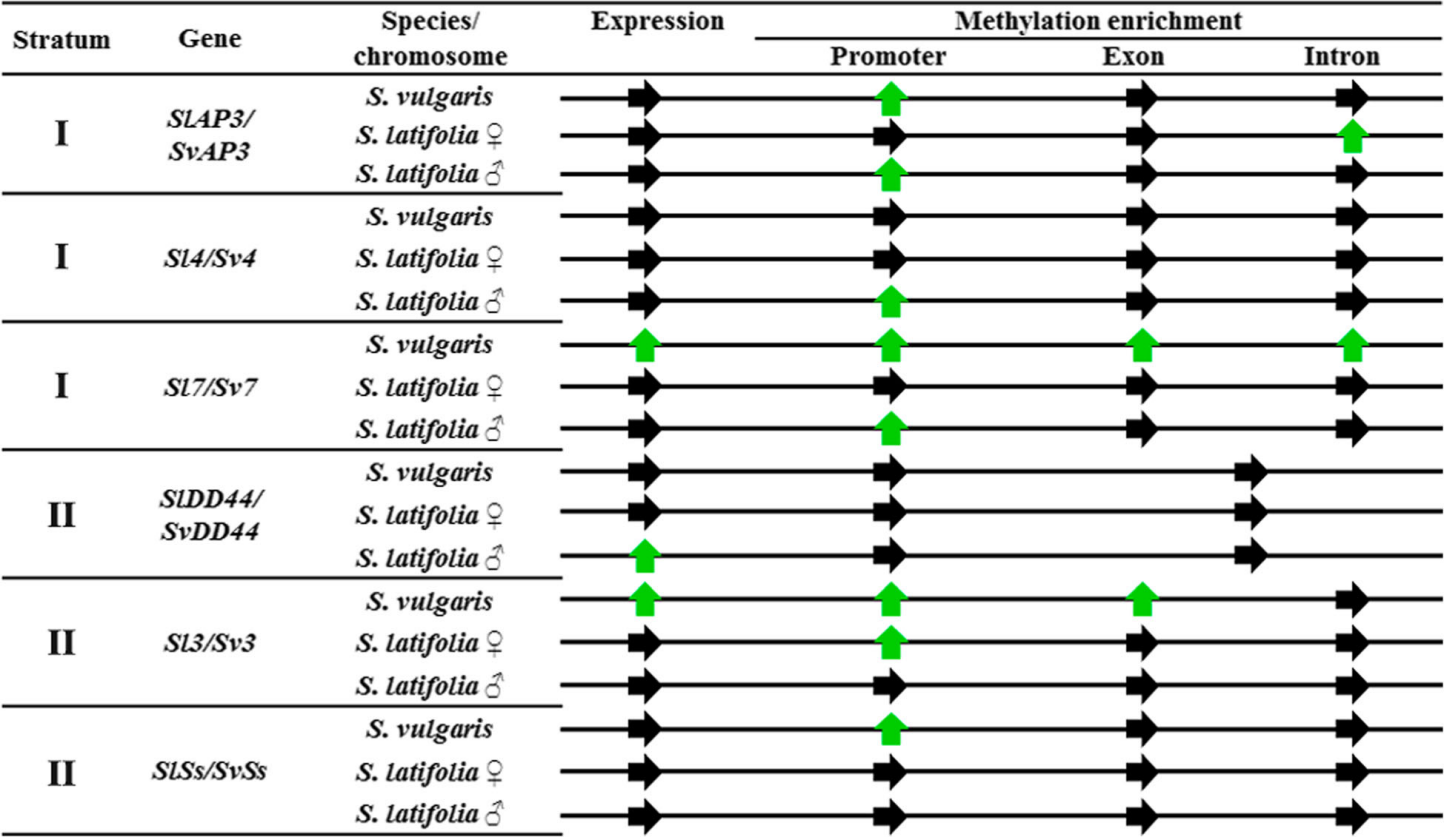
Schematic representation of gene expression and DNA methylation among *S. vulgaris* and *S. latifolia* male and female plants. Black arrows mean no significant difference. Green arrows mean differential expression and for methylation refers to significant methylation enrichment. Data for gene *DD44* includes exon and intron together (see Methods)

Comparison of the expression for *S. latifolia* and *S. vulgaris*, the correlation plot shows a high correlation between *S. latifolia* male and *S. vulgaris* (R^2^ = 0.705) and between *S. latifolia* female and *S. vulgaris* (R^2^ = 0.738). Expression data for the the vegetative stage revealed statistically significant differences between *S. latifolia* (male and female) and *S. vulgaris* on genes *Sl3* and *Sl7*. The differences between *S. latifolia* male and female were found only in *SlDD44*. In *S. latifolia* males, the expression was biased on both alleles, X and Y (Fig. 2). The expression was biased towards the X allele in *Sl7*, *SlDD44*, *Sl3* and *SlSs*. On the other hand the expression was biased towards the Y allele in *Sl4* and *SlAP3*.

### Sequence similarity and location

The degree of similarity of promoter and intron regions in *S. vulgaris*, the X allele of *S. latifolia* and Y allele of *S. latifolia*, was calculated for each gene by neighbour-joining tree nucleotide alignment (Additional file 2). The greatest similarity between X and Y alleles was found in the *SlDD44* intron region (91.13%) and the smallest in the *Sl7* promoter region (42.8%). For the rest of the sequences, the similarity ranged from 50 to 70%.

Regarding the location of the different genes, for the analysis, we used the sequence from the clones BAC30L22 and BAC241I12 [24, 25]. The genes *Sl7* and *SlAP3* (stratum I), are included in these clones and we found that in their proximity (± 1 Kb) there are TEs and repetitive sequences, but no other genes were found close to *Sl7* and *SlAP3*.

### Methylation in vegetative leaves

#### DNA immunoprecipitation and bisulfite sequencing

DNA methylation was analysed through MeDIP for three different regions of the genes described in the expression section. These were promoters, first exons and first introns (Fig. 2). The main differences were found in promoter regions. In *S. vulgaris*, methylation enriched promoters were found in *SvAP3*, *Sv7*, *Sv3* and *SvSs*. However, in *S. latifolia* male plants, there was only enrichment in genes from stratum I (*SlAP3*, *Sl4* and *Sl7*) and no enrichment in genes belonging to the stratum II. Regarding the X and Y alleles in *S. latifolia* males, the methylation enrichment on the stratum I genes, was only in the Y allele. Strikingly, only two genes were enriched in their intron region in *S. vulgaris Sv7* and in *S. latifolia* female plants *SlAP3*.

**Fig. 3:**
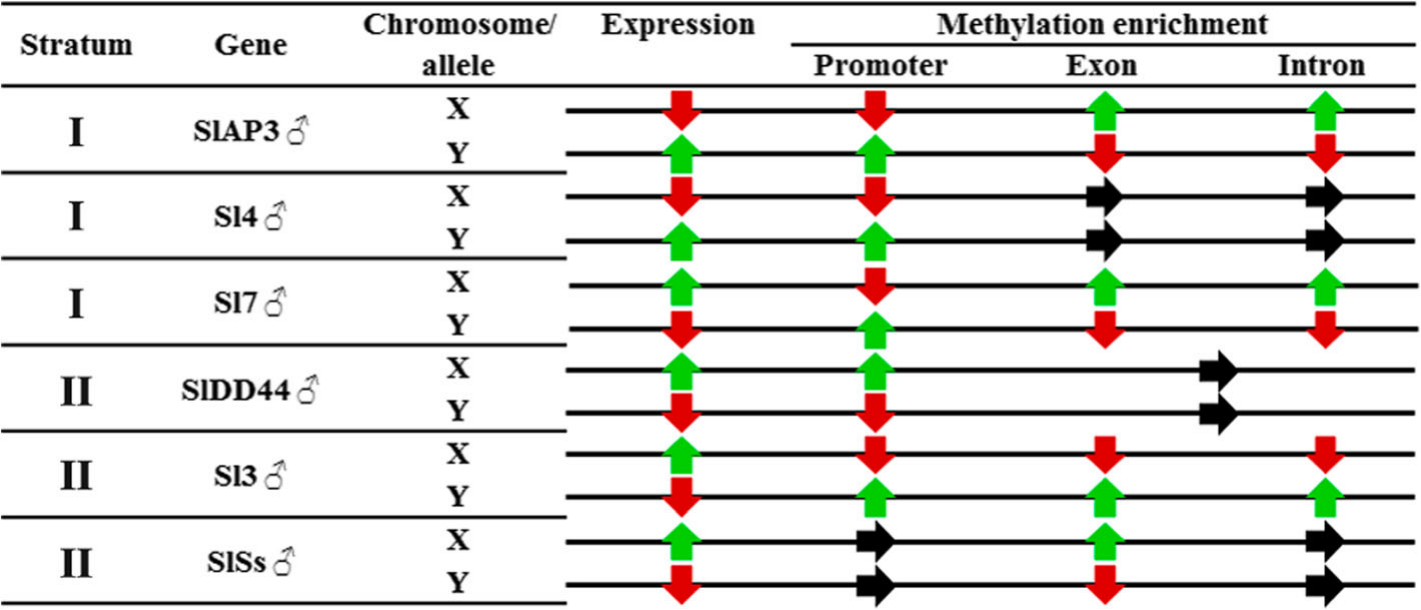
Schematic representation of gene expression and DNA methylation forn X and Y alleles in male plants. Black arrows - no significant difference. Green arrows- differential overexpression and for methylation refers to significant methylation enrichment. Red arrows mean differential underexpression and for methylation refers to non-significant methylation enrichment Data for gene *DD44* includes exon and intron together (see Methods)

**Fig. 4:**
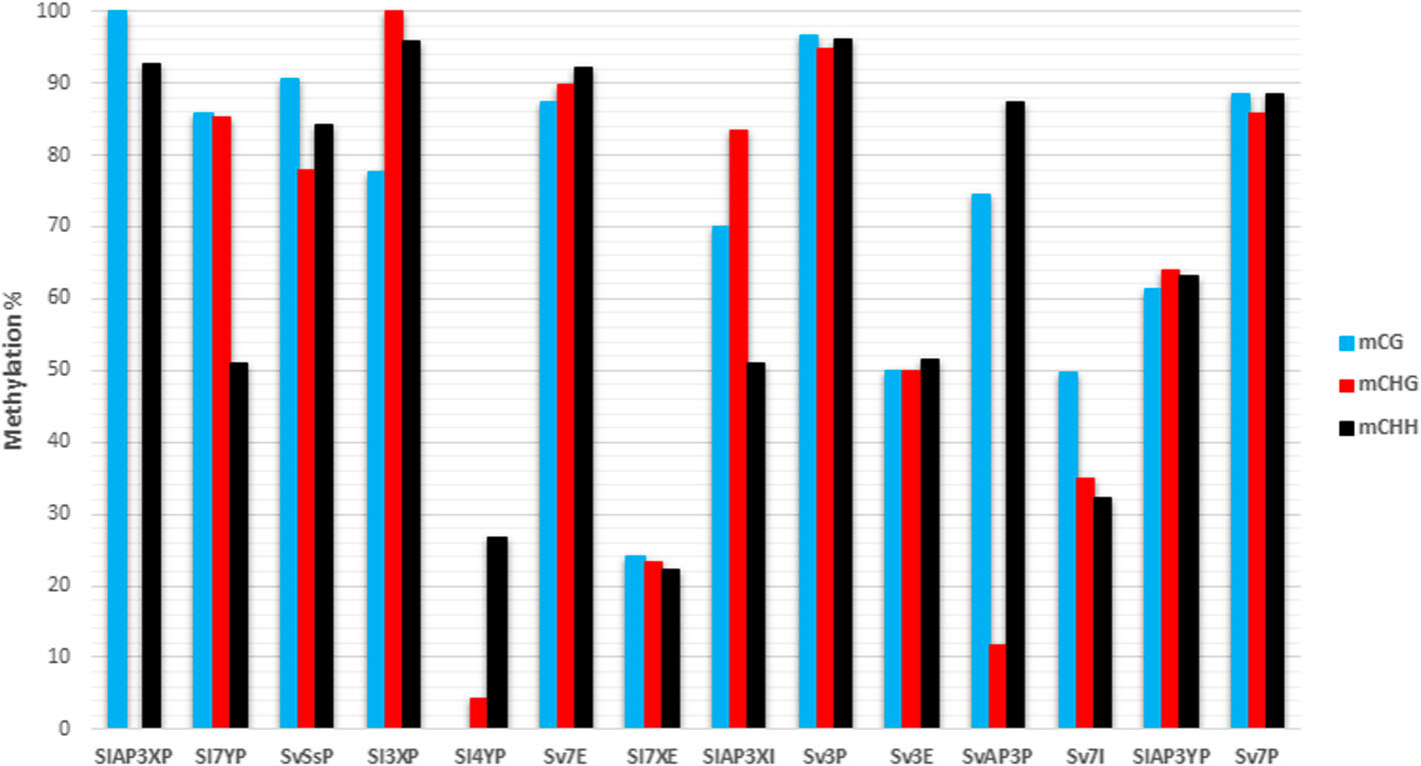
Relative % of CG, CHG and CHH methylation contexts in the different gene regions showing significant enrichment in the immunoprecipitation analysis. Gene name code: X; X allele, Y; Y allele, P; promoter, E; exon, I; intron

To support the results from MeDIP, Bisulfite sequencing was used on those sequences with significant enrichment. The results (Fig. 3; Additional file 3) showed no common pattern of methylation among genes belonging to the same stratum or in the same kind of region (promoter, exon or intron) in these genes. A close- up on individual genes, showed a similar percentage of methylation in most of the sequences analysed. From the three different methylation contexts in plants CG, CHG and CHH, the CHH context was the most abundant in all situations except for the sequences from gen *Sl7*; Sl7YP, Sv7E and Sl7XE (Fig. 4; Additional file 3). There was on average, over 50% methylation for this context in most sequences.

## Discussion

Most plant sex chromosomes appear to be of younger evolutionary origin than animal sex chromosomes and are, therefore, less degenerated [7, 26]. Degeneration of the genes analysed in this work, amongst others, has been analysed before by Marais [4] and Filatov [27]. Using whole genome analysis, rapid Y degeneration compared to the X allele has been reported by Papadopulos [26]. However, the latter found no significant differences in gene degeneration between evolutionary strata I and II. We need to bear in mind that these analyses were done on the coding sequence. In this study, we also performed a neighbour-joining tree nucleotide alignment test on promoter and intron regions. Even though we predicted that using the most divergent regions of the genes, there would be differences between strata I and II genes, no differences were found (Additional file 2). The similarity ranged from 50 to 70% in most cases. Given that variations in these sequences could affect methylation and therefore expression, the differences in methylation in the promoters of X and Y alleles may be influenced by modifications in the sequence per se. These modifications and not only sequences such as transposable elements and repetitive elements in the surroundings, may have a role in the differential methylation of both alleles [10, 19, 28].

Importantly, from the results of the MeDIP, only the promoters of genes from stratum I were differently methylated for male and female plants, suggesting a gene regulation difference between the strata. Either way, the methylation patterning males and females and also between X and Y alleles agree with the results in animals and some recent results in plants [18, 29]. The modification of DNA using sodium bisulfite which we used for methylation contexts, is the most widespread technique for analysing site-specific cytosine methylation [30]. Non CG context abundance and methylation are linked to repetitive and transposable elements [31]. Gehring [31] also indicated that a combination of CG and non CG contexts are related to gene silencing, though there are many exceptions. In most of the sequences analysed, the CHH methylation context is similar to CG and CHG contexts unlike other plants, which have a low methylation percentage in the CHH context [32, 33]. The CHH methylation context is, however, increased in TEs and repetitive sequences and their location are important for methylation spread [11, 32, 34]. Li [35] and Gent [36] have reported that TEs within 1 kb of genes have higher levels of CHH methylation and are enriched in 24 nt siRNA compared to genome wide averages. This suggests a regulation by RdDM. However, TEs that are in gene poor regions have a lower abundance of 24 nt siRNA and reduced CHH methylation levels suggesting that they are regulated by different mechanisms. The relation of RdDM to CHH methylation appears to be enriched when transposon insertion occurs near or within a gene to maintain the repression of the inserted TE. The specific influence of TEs on the expression of neighboring genes appears to vary with the type of transposable element and is also related to chromatin context [37]. De novo methylation, flanking intergenic chromatin 1 kb up and downstream of transcription start sites and transcription end sites correspondingly has been suggested and these regions have been labelled mCHH islands [38]. It has been suggested that these islands indicate the transition between heterochromatin-associated TEs and euchromatin-associated genes. In Arabidopsis CHH is correlated to CG and CHG methylation contexts. As a general trend CHH methylation was present several nucleotides downstream of methylated CG contexts, and four nucleotides downstream CHG contexts. Therefore, repeats rich in CG and CHG methylation might induce increased levels of CHH methylation in the described sequence context [39].

In spite of the large number of non CG contexts present in our sequences, a whole Y chromosome methylation analysis will be needed to determine if methylation, non CG context and the presence of repetitive elements are connected. Moreover the high percentage of methylation in Y promoters does not involve silencing in all genes. In maize, the loss of methylation in mCHH islands found in expressed genes that are located near TEs does not impact gene expression, suggesting that promoter methylation at CHH islands in maize may not simply control gene expression but may also regulate nearby cis elements [38].

## Conclusions

In summary, DNA methylation in genes under analysis in males was only found in the promoter of genes belonging to stratum I (older), of these genes, the Y alleles were more methylated than X alleles. There was high abundance and % methylation in the CHH context in most sequences. These two main conclusions suggest that the age of the stratum in the chromosome affects methylation of the genes in the stratum. The position in the chromosome also has an influence on methylation as well as the proximity of TEs and repetitive sequences. We speculate that TE accumulation and not gene degeneration is the cause of high DNA methylation in *S. latifolia* Y sex chromosomes with influence on the heterochromatinisation of the chromosome.

## Methods

### Plant material

All species of the genus *Silene* used in this study came from the collection of the Institute of Biophysics in Brno, Czech Republic. An inbred population (U15; 15 generations of brother x sister cross) of male and female *S. latifolia* was used in this research. *Silene vulgaris*, a close non-dioecious relative without sex chromosomes, was used as an out-group. Plant material was grown in a cultivation room under standard conditions (t 24uC, 16 h light/8 h dark).

### Whole gene sequence characterization

Full-length gene characterization was performed using the information available on the databases from the bacterial artificial chromosome (BAC) library characterized by Blavet [40]. We also used the information provided by the NGS data from [26, 41]. Sequence management was done using Geneious v7.1 (Biomatters, http://www.geneious.com). The remaining unknown sequences of the X and Y alleles or *S. vulgaris* copies were identified by SMARTer™ RACE cDNA Amplification Kit (coding sequence) and by GenomeWalker™ Universal Kit (promoter and intron sequence) both from Clontech (Mountain View, CA, USA). All the primers used in the characterization of the different sequences are listed in Additional file 4: Table S1. Total DNA and RNA were isolated from leaves and they were used as a template for the amplification reactions. Genomic DNA was isolated from young leaves using DNAeasy Plant Mini Kit (Qiagen, Dusseldorf, Germany). RNA was isolated from 75 mg of frozen tissue according to RNeasy Plant Mini Kit (Qiagen, Dusseldorf, Germany). RNA integrity was tested in agarose gel under denaturing conditions. Removal of primers and nucleotides from PCR products was carried out through ExoSAP. Samples were Sanger sequenced in an ABI 3730xl device (Applied Biosystems, Foster City, CA, USA), after labelling (BigDye Terminator v3.1; Thermo Fisher Scientific, Houston, USA) and purification (Agencourt® CleanSEQ®; Agencourt Bioscience Corporation, Beverly, MA).

### Real time expression

Quantitative PCR was performed as follows: 10 ng of cDNA, 5 μM of each primer and SensiFAST HRM Kit (Bioline Reagents Ltd., London, UK) were mixed and amplified using the RotorGene Q (Qiagen, Dusseldorf, Germany). Three individual replicates of cDNA obtained using Superscript III first strand synthesis kit, (Invitrogen, Gaithersburg, MD, USA) were quantified, and data analysis was performed using the LinReg software [42]. GADPH and 18S were previously selected by GeNorm software [43], and were used as endogenous controls to calculate relative expression. Primer sequences and Tm are indicated in Additional file 4: Table S1.

The analysis of X and Y alleles in males was carried out as follows: primers were designed from a common region in each gene under analysis. The X and Y amplicons, which had SNPs between alleles, were cloned in pCR2.1 vector (TA Cloning, Invitrogen, Carlsbad, CA, USA) and X:Y ratios of 0:100; 25:75; 50:50; 75:25; 100:0 were used for the real time analysis. The differential expression of X and Y alleles was measured by High Resolution Melting analysis. Using the standard melt curve data, linear regression was used to calculate the X:Y expression ratio in *S. latifolia* male tissues.

### Methylated DNA Immunoprecipitation

#### DNA isolation

A fresh weight of 300 mg of *S. latifolia* vegetative leaves was used for DNA extraction by nuclei purification, as follows: a volume of 10 mL of Buffer A (0.44 M sucrose, 10 mM Tris-HCl; pH 8.0, 5 mM β-ME and 0.15 mM PMFS) was added to the frozen tissue powder and the samples were incubated in ice for 30 min with occasional shaking. Samples were filtered with a mousseline and centrifuged at 3000 g for 15 min at 4 °C. Several extractions with Buffer B (0.25 M sucrose, 10 mM Tris-HCl; pH 8.0, 10 mM MgCl2, 1% Triton X-100, 5 mM β-ME and 0.15 mM PMSF) were used to remove organelles until the pellet was mostly white. It was resuspended in 8 mL of buffer C (0.25 M sucrose, 10 mM Tris-HCl; pH 8.0, 10 mM MgCl2, 5 mM β-ME and 0.15 mM PMSF) and centrifuged at 3000 g for 10 min at 4 °C. Nuclei were re suspended in 1 mL of nuclei lysis buffer (50 mM Tris-HCl; pH 8, 10 mM EDTA, 1% SDS and 15 μM PMSF). Samples were phenol/chloroform purified and DNA was ethanol precipitated and quantified.

### DNA sonication

An amount of 20 μg of genomic DNA was diluted in 300 μL of IP buffer (100 mM Na-phosphate; pH 7.0, 1.4 M NaCl and 0.5% Triton X-100) in a 1.5 mL tube. A total of 7 pulses of 90% intensity for 15 s for each sample at 4 °C were delivered with an ultrasonic processor UP50H (Hielscher Ultrasonics GmbH, Teltow, Germany). Verification of the size of the fragment of sonicated DNA on a 2% agarose gel was carried out with an average size of 400 bp and a range of from 200 to 800 bp. A total of 14 μg of DNA was placed in two 1.5 mL tubes (7 μg of DNA per tube).

### Immunoprecipitation of methylated DNA

Prior to the immunoprecipitation protocol, internal methylated and unmethylated standards were added to each sample for evaluation of immunoprecipitation performance (Diagenode, Seraing, Belgium). Samples were heat-denatured and immediately placed in ice for 10 min. An aliquot of each sample was stored at − 20 °C as an input control. An amount of 10 μg of antibody (monoclonal mouse anti-5-methylcytidine. Diagenode, Seraing, Belgium) was added to each sample. The mixture was incubated on a rotating platform at 4 °C. Then to the DNA-antibody mixture, a volume of 50 μl of magnetic Dynabeads® Protein G (Invitrogen Life Technologies Corporation, Gaithersburg, MD, USA) was added. The samples were later incubated for 2 h on a rotating platform at 4 °C. A magnetic rack (Invitrogen Life Technologies Corporation, Gaithersburg, MD, USA) was used to remove the supernatant and for the washing steps. In total we performed three washes and later on we resuspended the magnetic beads in 250 μl of digestion buffer (50 mM Tris; pH 8.0, 10 mM EDTA and 0.5% SDS) with 100 μg of Proteinase K (Sigma Chemical Co., St. Louis, MO, USA). The digestion with Proteinase K was realized in a hybridization oven with rotation at 50 °C.

### Purification of methylated DNA

Samples were phenol extracted (phenol:chloroform:isoamyl alcohol (*v*/v 25:24:1)) in 2 ml heavy 5 prime phaselock tubes (Eppendorf-5 Prime, Inc., Boulder, CO, USA). DNA was ethanol precipitated overnight at − 80 °C using 1.5 μl glycogen (20 mg/mL) as an adjuvant. After centrifugation (20,000 g for 10 min at 4 °C), the pellet was resuspended in TE buffer and quantified.

### Quantitative PCR

Quantitative PCR was performed as follows: 10 ng of immunoprecipitated DNA, 5 μM of each primer and SensiFAST HRM Kit (Bioline Reagents Ltd., London, UK) were mixed according to manufacturer’s instructions and amplified using the Rotor-Gene Q (Qiagen, Dusseldorf, Germany). Three regions from each gene were selected for amplification analysis, promoter region (500 bp upstream from transcription starting site), first exon and first intron. The exception was gene *SlDD44* which has a first exon of 80 bp. Given that recommended amplicon size should be over 100 bp, in this gene we used for amplification the region including the first exon, first intron and second exon. Four replicates for each transcript from a pool of individuals were analysed using the LinReg software [42]. Primer sequences and Tm are indicated in Additional file 4: Table S1.

### Statistical analysis

Univariate analysis of variance (ANOVA) was carried out on all EST data using the glm procedure in R Studio (V1.0.143) to identify factor interactions. Multiple comparisons were then made with the post hoc Tukey’s HSD test. Single comparisons were made using Student *t*-tests.

### Single cytosine resolution methylation

The gene regions with significant differences in methylated DNA immunoprecipitation were selected for further analysis by bisulfite sequencing. DNA was converted using EZ DNA Methylation™ Kit (Zymo Research, California, USA). For amplification of the target sequence by PCR, modified primers using Bisprimer [44] and Methyl Primer Express v1.0 (Applied Biosystems, Foster City, CA, USA) were designed. According to V Kovacova and B Janousek [44], a nonselective variant primer (Nv) which is able to bind methylated and no methylated modified DNA templates with the same efficiency, was designed. The amplified products were cloned in pCR2.1 vector (TA Cloning, Invitrogen, Carlsbad, CA, USA) and Sanger sequenced (Macrogen Ltd., Seoul, South Korea). The sequencing data of 30 clones from each amplification was analysed using Kismeth [45].

## Additional files

Additional file 1: Expression and methylation enrichment data. This is an excel file with the expression and methylation graphs belonging to the different genes under analysis in this work. There is also included an expression correlation plot between *Silene latifolia* and *Silene vulgaris*. (XLSX 156 kb)

Additional file 2: Gene sequence similarity. This is an excel file including the percentage of similarity when comparing the same region (e.g. promoter) of a gene in *Silene vulgaris* and in *Silene latifolia* X and Y alleles. (XLSX 16 kb)

Additional file 3: Bisulfite sequencing analysis. This is an excel file with the data of sodium bisulfite sequencing analysis from those regions in the different genes with a significant enrichment in the MeDIP experiment. These data were obtained to support the positive results in the MeDIP to confirm the methylation enrichment. A general methylation context plot is also included. (XLSX 16980 kb)

Additional file 4: **Table S1.** Primer list. List of primers used in this work. (XLSX 19 kb)

## Abbreviations

MeDIP: Immunoprecipitation of methylated DNA
PAR: Pseudoautosomal region
RdDM pathway: RNA directed DNA methylation pathway
SPF: Stamen promoting function
